# Supplementary figures and images for: Cranial anatomy of Besanosaurus leptorhynchus Dal Sasso & Pinna, 1996 (Reptilia: Ichthyosauria) from the Middle Triassic Besano Formation of Monte San Giorgio, Italy/Switzerland: taxonomic and palaeobiological implications
Source: PeerJ. 2021 May 6;9:e11179. doi: 10.7717/peerj.11179 (PMC8106916; doi:10.7717/peerj.11179)

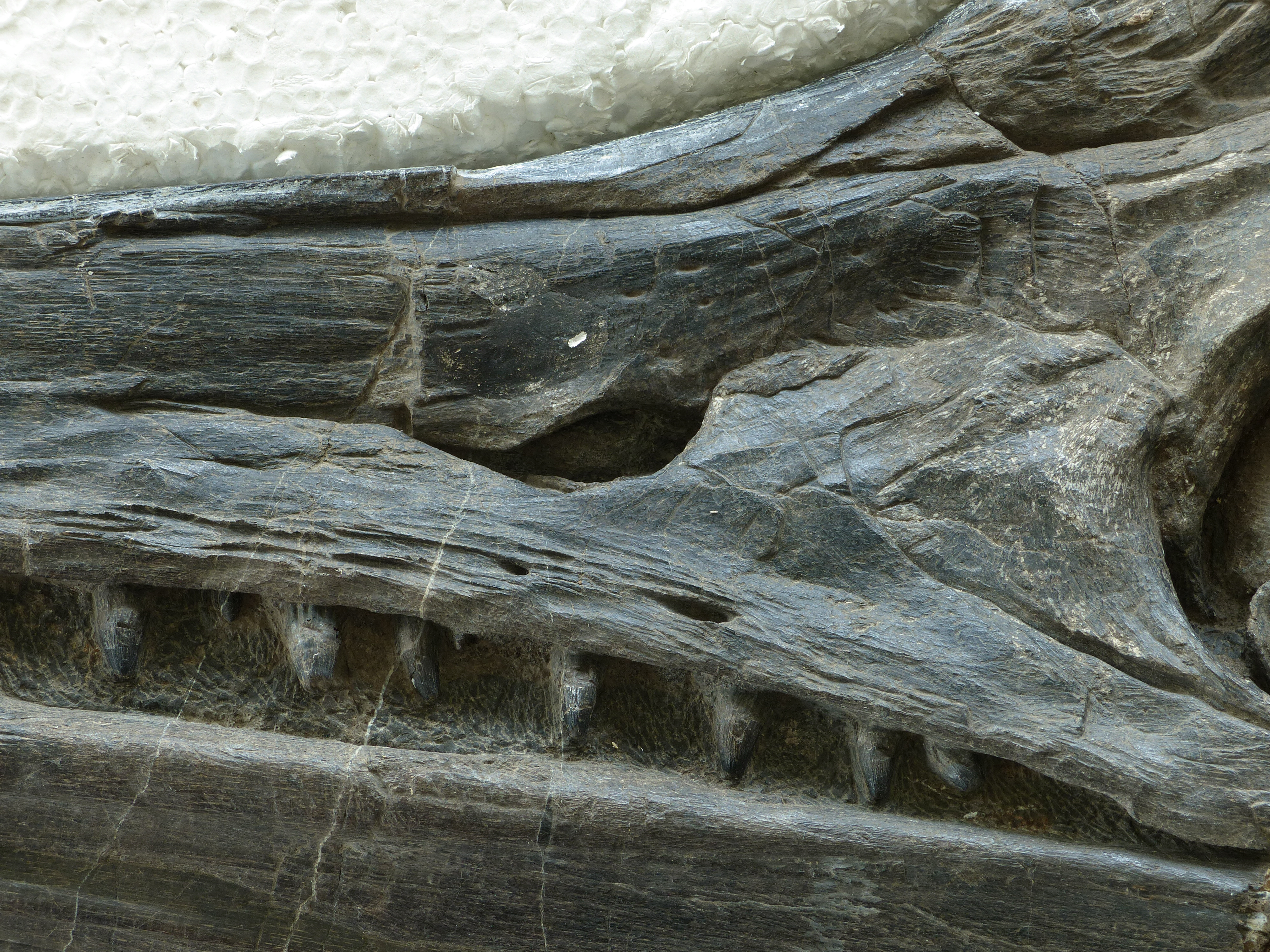

Supplement: Supplemental Information 1 — Perinarial region of a 3D-preserved referred specimen of Guizhouicthyosaurus tangae (IVPP V11869) in left lateral view. For bone interpretation, see Li & You (2002: fig. 2). Scale bar represents 5 cm. [file peerj-09-11179-s001.jpg]

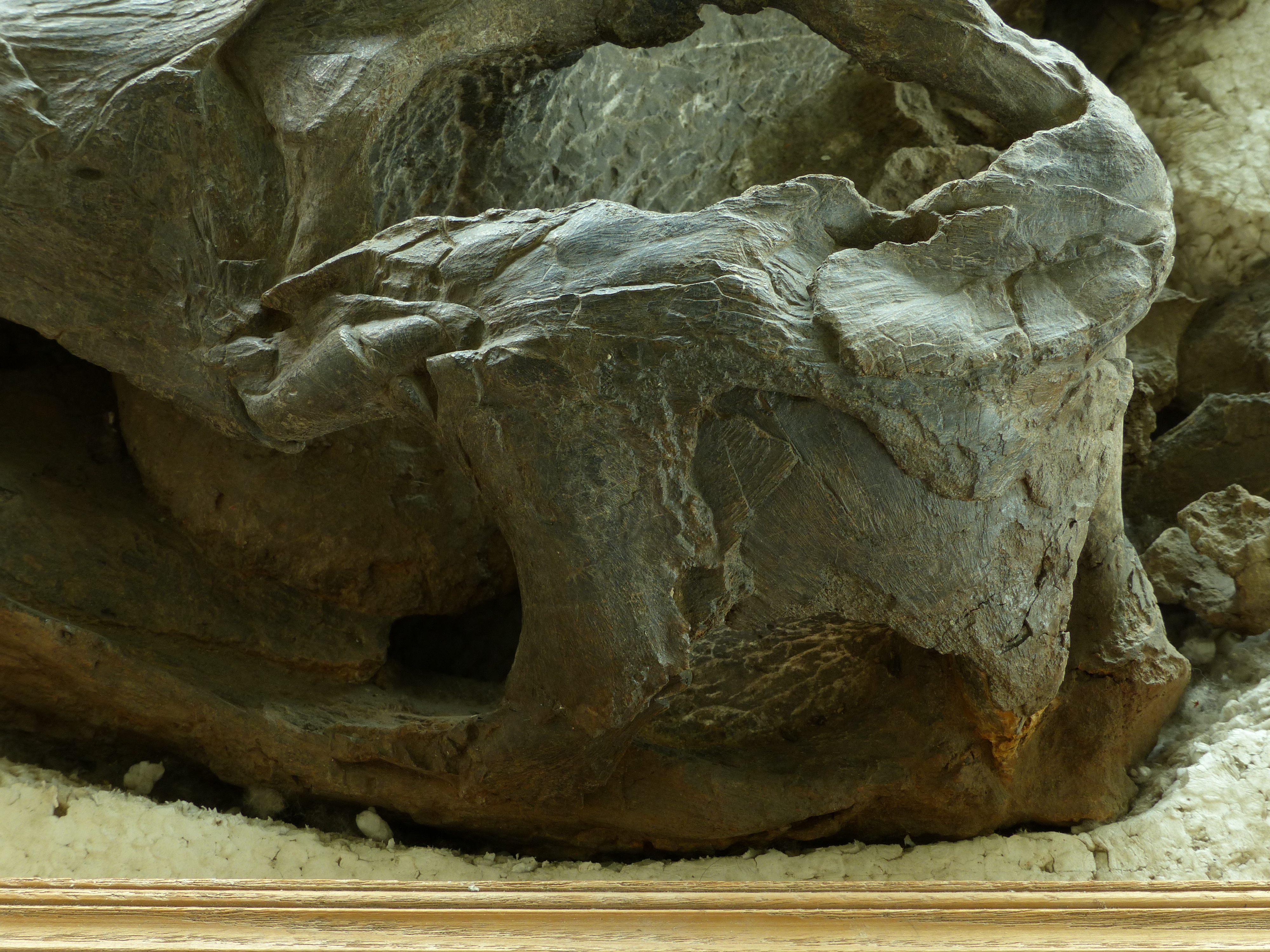

Supplement: Supplemental Information 2 — Postorbital region of a 3D-preserved referred specimen of Guizhouicthyosaurus tangae (IVPP V11865) in left lateral view. Abbreviations: see text. Scale bar represents 5 cm. [file peerj-09-11179-s002.jpg]

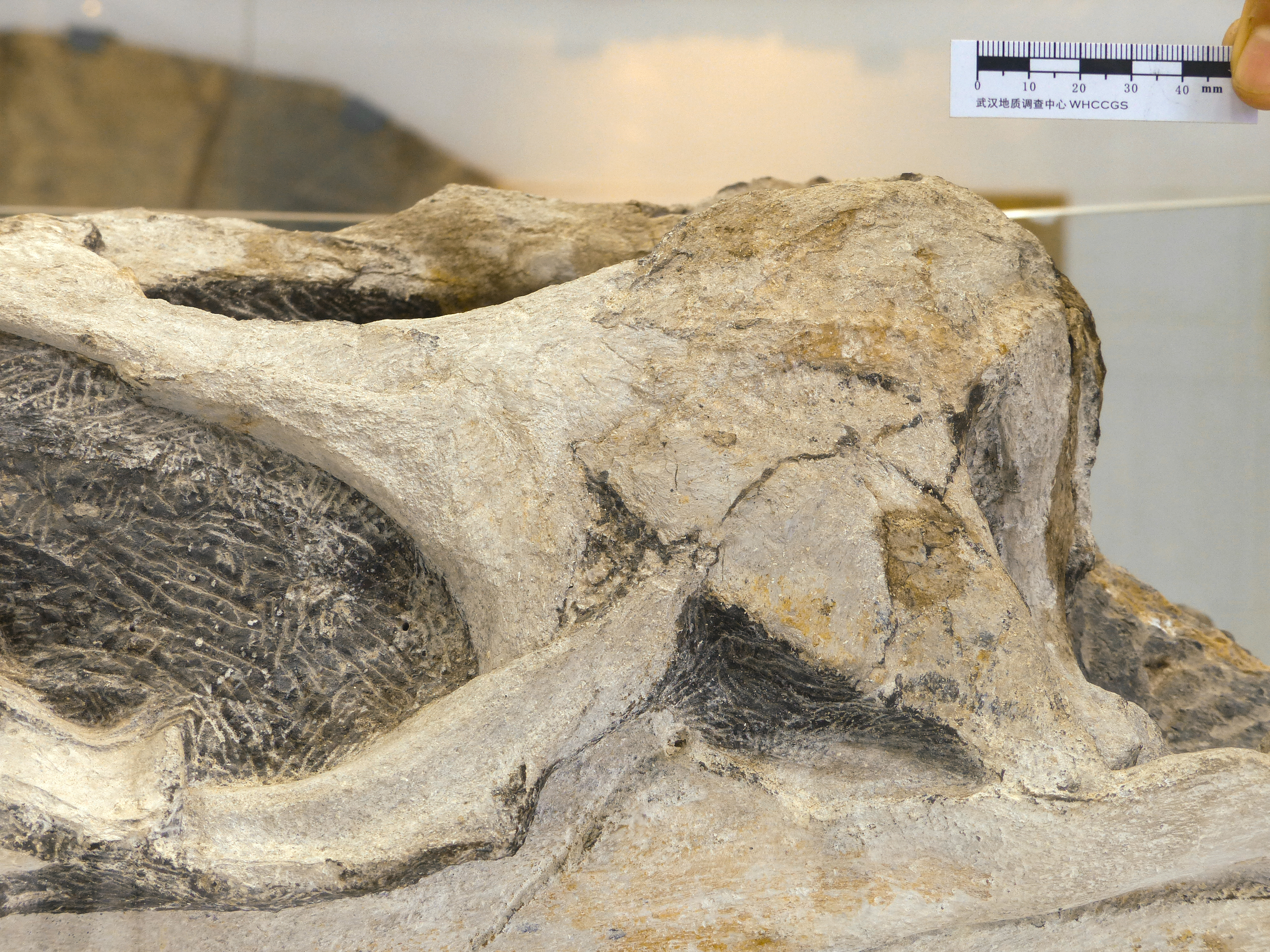

Supplement: Supplemental Information 3 — Postorbital region of a 3D-preserved referred specimen of Guanlingsaurus liangae (SPCV 03107) in left lateral view. For bone interpretation, see Sander et al. (2011: fig. 2b). [file peerj-09-11179-s003.jpg]

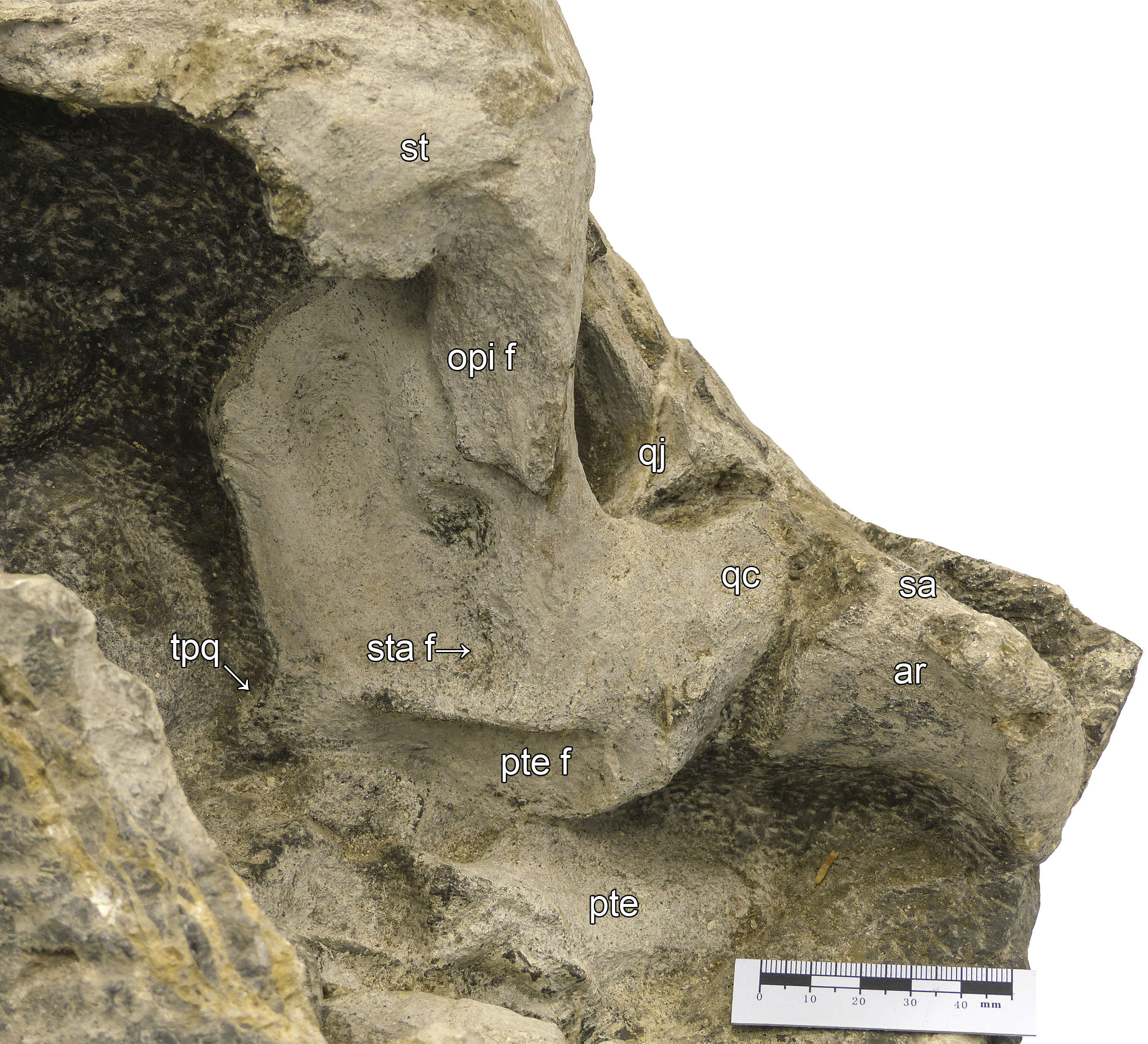

Supplement: Supplemental Information 4 — Occipital region of a 3D-preserved referred specimen of Guanlingsaurus liangae (SPCV 03107) in caudomedial view. Note the presence of the triangular process of the quadrate, which helps to hold the caudolateral flange of the pterygoid. Abbreviations: see text. Scale bar represents 5 cm. [file peerj-09-11179-s004.png]

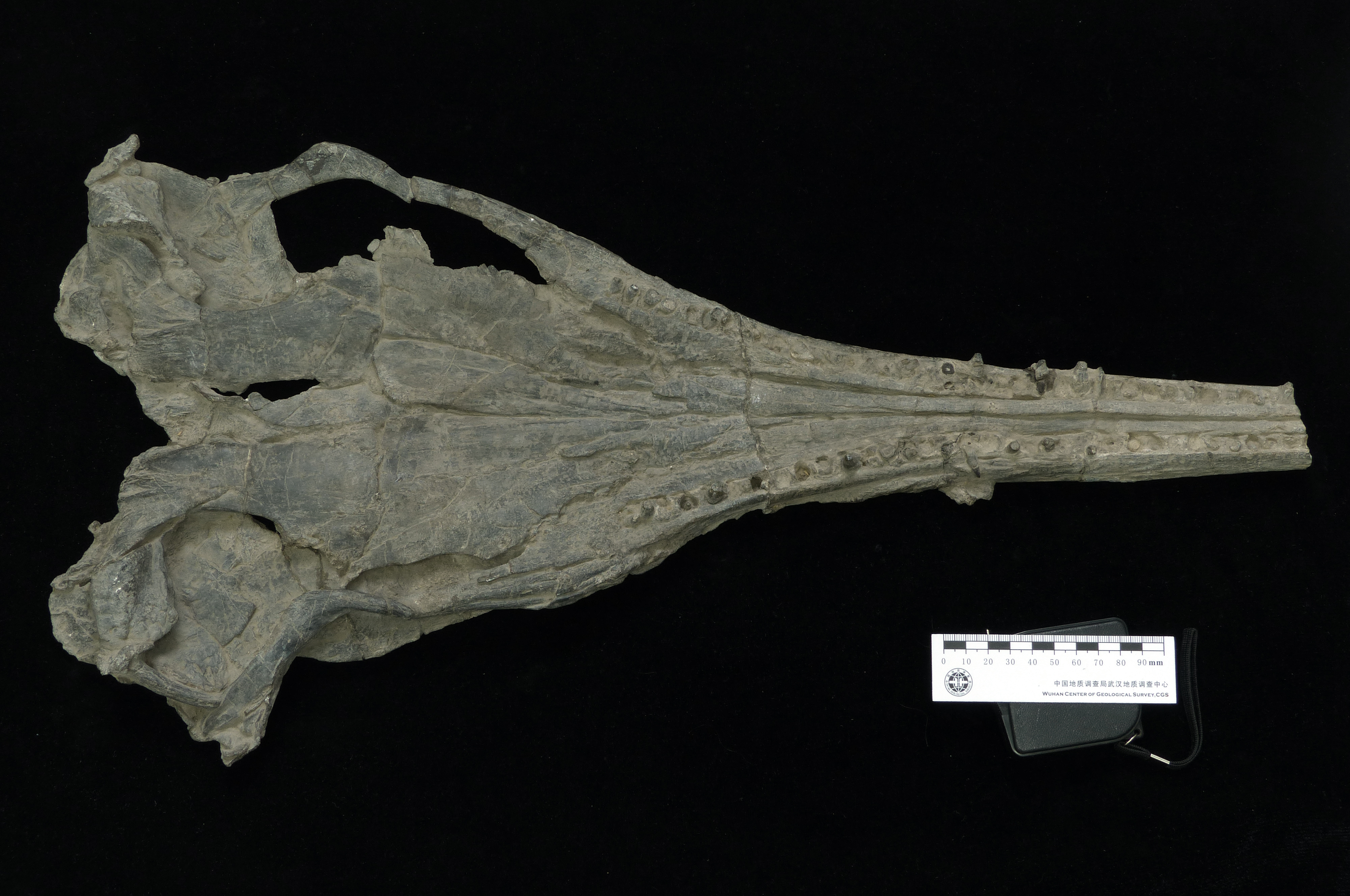

Supplement: Supplemental Information 5 — Palatal view of a referred specimen of ‘Callawayia’ wolonggangense (SPCV 10305). For bone interpretation, see Chen et al. (2007: fig. 2D). [file peerj-09-11179-s005.jpg]

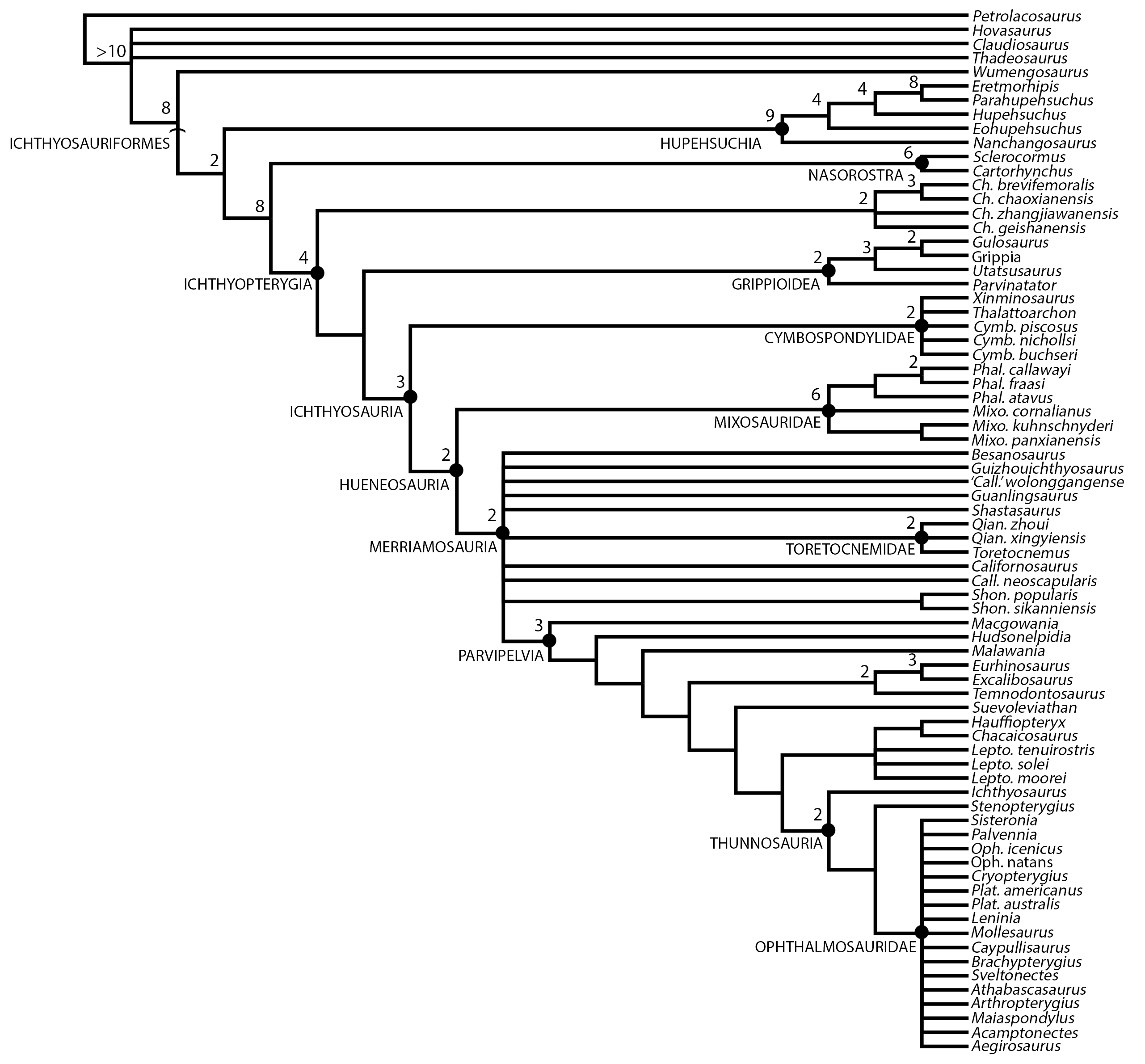

Supplement: Supplemental Information 6 — Strict consensus of 14,480 MPTs of 713 steps (CI=0.363, RI=0.788) obtained from parsimony analysis of the character-taxon matrix of Huang et al. (2019). Note large polytomy at the base of Merriamosauria. Numbers above nodes indicate Bremer support values. Abbreviations: Call., Callawayia; Ch., Chaohusaurus; Cymb., Cymbospondylus; Lepto., Leptonectes; Mixo., Mixosaurus; Oph., Ophthalmosaurus; Qian., Qianichthyosaurus; Phal., Phalarodon; Shon., Shonisaurus. [file peerj-09-11179-s006.png]
