# Supplementary material for: Cranial anatomy of Besanosaurus leptorhynchus Dal Sasso & Pinna, 1996 (Reptilia: Ichthyosauria) from the Middle Triassic Besano Formation of Monte San Giorgio, Italy/Switzerland: taxonomic and palaeobiological implications
Source: PeerJ. 2021 May 6;9:e11179. doi: 10.7717/peerj.11179 (PMC8106916; doi:10.7717/peerj.11179)
Supplement: Supplemental Information 7 — 3D model obtained with photogrammetry of the skull and mandible of BES SC 999; 3D model obtained with photogrammetry of the skull and mandible of PIMUZ T 4376; 3D model obtained with photogrammetry of specimen GPIT 1793/1. [file peerj-09-11179-s007.docx]

Google drive links to download the 3D file:

**File S1.** 3D model obtained with photogrammetry of the skull and mandible of BES SC 999.

<https://drive.google.com/file/d/1g9lDN0SFol9C6c4zGSgoBf3grVKi0uoj/view?usp=sharing>

**File S2.** 3D model obtained with photogrammetry of the skull and mandible of PIMUZ T 4376.

<https://drive.google.com/file/d/1dLv4mcsehxo3dom9nB1fA8vyRDILomg2/view?usp=sharing>

**File S3.** 3D model obtained with photogrammetry of specimen GPIT 1793/1.

<https://drive.google.com/file/d/1dUGigFPtx3WnJ3uepGH6mF8RFtM-H7oP/view?usp=sharing>

Open the files with the latest version of Adobe Reader.
